# Targeting NR1D1 in organ injury: challenges and prospects

**DOI:** 10.1186/s40779-023-00495-3

**Published:** 2023-12-11

**Authors:** Zi-Yin Zhang-sun, Xue-Zeng Xu, Germaine Escames, Wang-Rui Lei, Lin Zhao, Ya-Zhe Zhou, Ye Tian, Ya-Nan Ren, Darío Acuña-Castroviejo, Yang Yang

**Affiliations:** 1 Department of Cardiology, Northwest University First Hospital, Faculty of Life Sciences and Medicine, Northwest University, Xi’an, 710069 China; 2 Key Laboratory of Resource Biology and Biotechnology in Western China, Ministry of Education, Faculty of Life Sciences and Medicine , Northwest University, Xi’an, 710069 China; 3 Department of Cardiovascular Surgery, Xijing Hospital, Air Force Medical University, Xi’an, 710032 China; 4 Biomedical Research Center, Department of Physiology, Faculty of Medicine, Institute of Biotechnology, Technological Park of Health Sciences, University of Granada, 18016 Granada, Spain; 5 Centro de Investigación Biomédica en Red Fragilidad y Envejecimiento Saludable (CIBERFES), Ibs.Granada, San Cecilio University Hospital, 18016 Granada, Spain; 6 UGC of Clinical Laboratories, San Cecilio Clinical University Hospital, 18016 Granada, Spain

**Keywords:** NR1D1, REV-ERBα, Circadian rhythms, Liver, Heart, Lung, Kidney

## Abstract

Nuclear receptor subfamily 1, group D, member 1 (NR1D1, also known as REV-ERBα) belongs to the nuclear receptor (NR) family, and is a heme-binding component of the circadian clock that consolidates circadian oscillators. In addition to repressing the transcription of multiple clock genes associated with circadian rhythms, NR1D1 has a wide range of downstream target genes that are intimately involved in many physiopathological processes, including autophagy, immunity, inflammation, metabolism and aging in multiple organs. This review focuses on the pivotal role of NR1D1 as a key transcription factor in the gene regulatory network, with particular emphasis on the milestones of the latest discoveries of NR1D1 ligands. NR1D1 is considered as a promising drug target for treating diverse diseases and may contribute to research on innovative biomarkers and therapeutic targets for organ injury-related diseases. Further research on NR1D1 ligands in prospective human trials may pave the way for their clinical application in many organ injury-related disorders.

## Background

Nuclear receptor subfamily 1, group D, member 1 (NR1D1, also known as REV-ERBα) was first discovered in 1989 and is an approximately 56 kD protein encoded by the ERBA (also known as THRA) oncogene’s reverse DNA strand [1]. In 1994, multiple labs successfully discovered a new orphan receptor with high homology to the rat REV-ERBα gene product (especially in the DNA binding domain and ligand binding domain) referred to as NR1D2 or REV-ERBβ [2–4]. NR1D1 and NR1D2 are key transcriptional repressors of regulatory networks with a circadian expression pattern and are widely expressed in many tissues [1, 5].

NR1D1 can bind to the promoters of many genes to mediate transcriptional repression of autophagy-associated proteins, inflammasome genes, T-cell differentiation cofactors, lipid-metabolizing enzymes, and other important players in the physiological and pathological processes of various organs. Molecularly, NR1D1 inhibits enhancer-derived RNA (eRNA) transcription and reduces target gene mRNA expression by recruiting the nuclear receptor corepressor (NCOR)-histone deacetylase 3 (HDAC3) complex to the enhancers or promoters of target genes [6].

This review examined the milestones of the latest discoveries regarding NR1D1 in many physiopathological processes in multiple organs, including the liver [7], heart [8], lung [9], and kidney [10]. According to NR1D1 involvement in autophagy, immunity, inflammation, aging and metabolism, researchers have identified and designed diverse natural and synthetic NR1D1 ligands that are capable of stimulating or blocking inherent signal transduction. These agonists and antagonists are typically small synthetic compounds, and some have progressed to preclinical trials. Therefore, NR1D1 is considered a prospective pharmacological target of numerous diseases, and it may contribute to providing novel insights into therapeutic strategies for organ injury.

## The structure and action forms of NR1D1

NR1D1 and NR1D2 belong to the nuclear receptor (NR) subfamily, but their structures are slightly different from the classical nuclear receptors [11, 12]. There are four primary domains that distinguish typical nuclear hormone receptors in NRs: a variable amino N-terminal activation function 1 (AF-1), a highly conserved DNA-binding domain (DBD) consisting of two zinc finger motifs, a hinge region linking the DBD to the carboxy-terminal ligand-binding domain (LBD), and a conserved LBD mediating coactivator interactions through the absence or presence of regulatory AF-2 region [13, 14]. DBD facilitates the precise recruitment of NRs monomers, homodimers, and heterodimers to their DNA response element after targeting the receptor to certain DNA sequences known as hormone response elements. Along with the hinge region and the LBD, DBD also participates in the dimerization of NRs with their partner. Additionally, LBD promotes ligand-dependent interactions with transcriptional co-activators or co-repressors through conformational changes. After binding ligand, LBD allows the receptor to switch into a transcriptionally active state. It also has the crucial feature of hormone recognition and controls the selectivity and specificity of the physiologic response [15]. These half-sites are arranged either as a palindromic sequence or a direct repeat. Carboxyl terminal helix AF-2 segment identifies coactivators necessary for transcriptional activation, and is crucial for ligand-dependent recruitment of coactivators and NRs transcriptional activation. Notably, NR1D1 and NR1D2 lack AF-2 region, and are therefore considered to be incapable of activating transcription. Hence, they are indeed constitutive transcriptional inhibitors that control the transcription of genetic information by binding to specific DNA sequence.

**Fig. 1:**
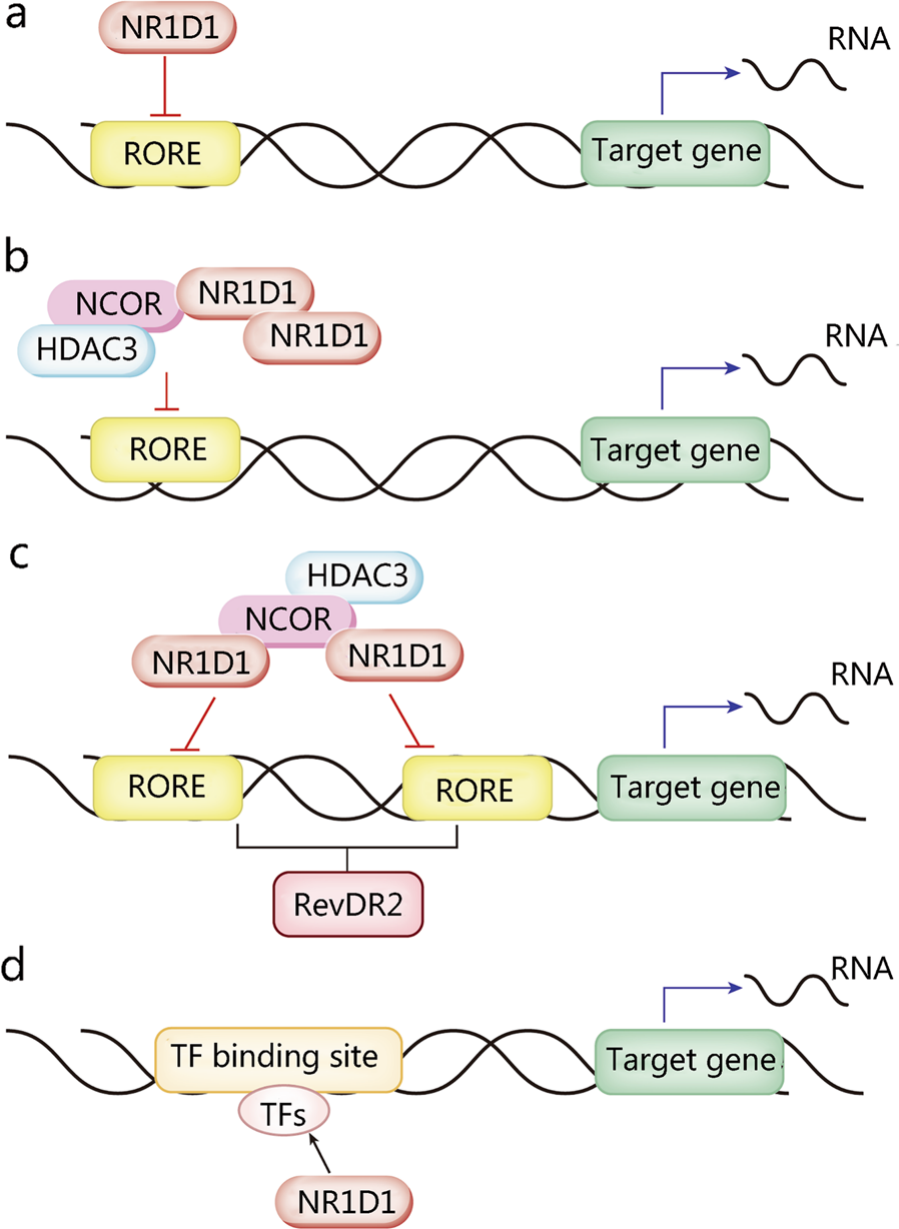
Four transcriptional inhibitory modes of NR1D1. **a** NR1D1 can bind to a single RORE as a monomer but cannot recruit the co-repressor NCOR1-HDAC3; **b** NR1D1 can bind to a RORE as a homodimer and recruit the co-repressor NCOR1-HDAC3; **c** Two NR1D1 monomers can also recruit NCOR1-HDAC3 to repress transcription when they bind independently to two ROREs; **d** NR1D1 can also coordinate transcriptional repression together with TFs. HDAC3 histone deacetylase 3, NCOR nuclear receptor corepressor, NR1D1 nuclear receptor subfamily 1, group D, member 1, RORE ROR response element, TF transcription factor

**Fig. 2:**
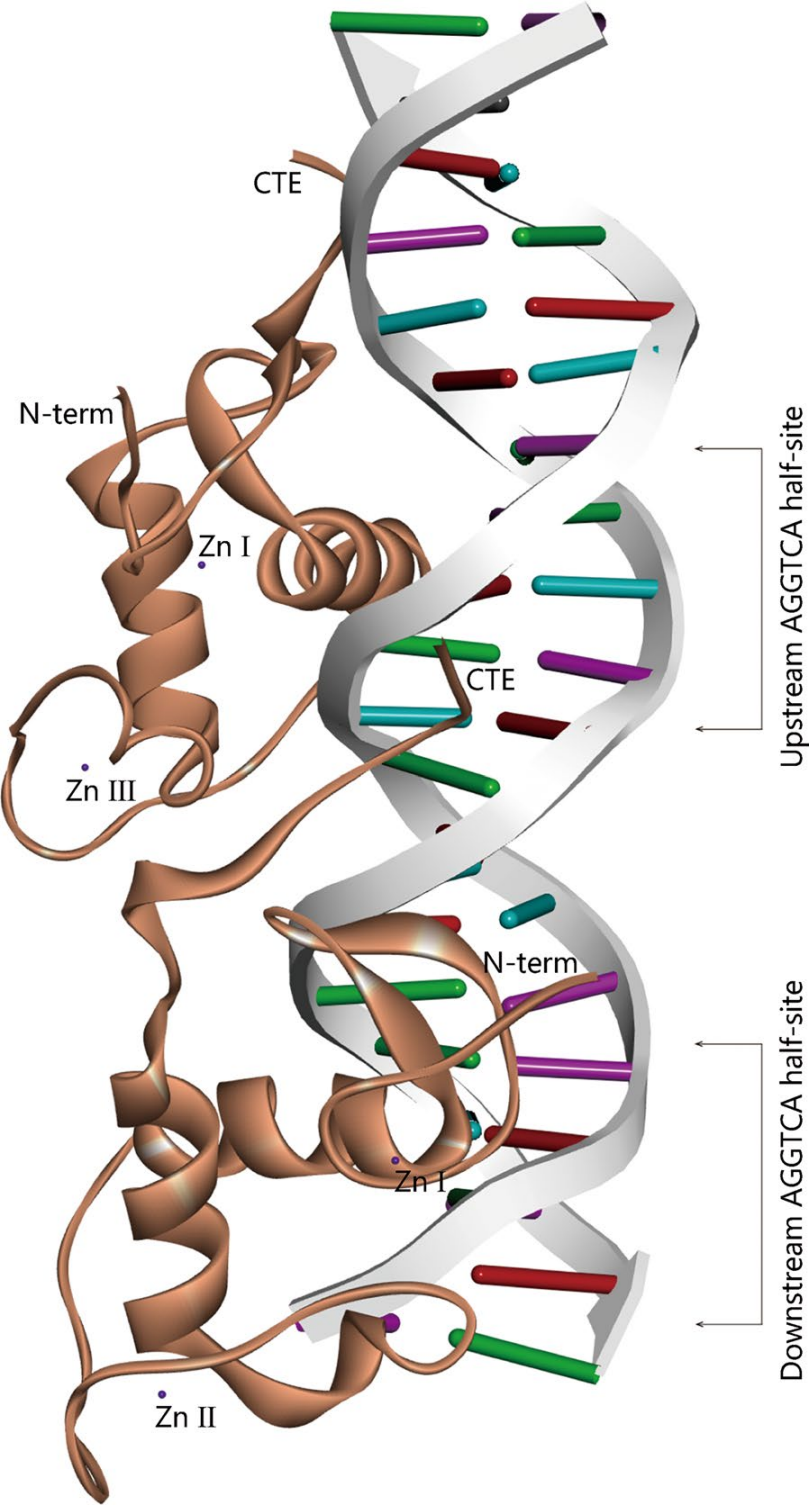
NR1D1 action structure domain. Two NR1D1 respectively bind to the AGGTCA half-site of the DNA sequence through DBD domain. CTE C-terminal, DBD DNA-binding domain

NR1D1 has two main action forms to suppress transcription, that is monomer and homodimer (Fig. 1). NR1D1 generally acts as a monomer to bind to the thyroid/retinoic acid receptor half-site AGGTCA, which is flanked 5’ by an A/T-rich sequence. This half-site is located in target genes promoter, and is referred to ROR/REV-ERB-response element (RORE/RevRE) [2, 16]. In 1998, Zhao et al. [17] revealed the interaction between the A/T-rich 5’ extension of the AGGTCA half-site and the C-terminal extension of DBD enhances their high affinity. In addition, NR1D1 acts as a homodimer to bind to tandem repeat sequences of Rev monomer sites spaced by 2 bp (“DR2”), while the A/T-rich sequences flanks on the 5’ half-site of DR2. Such that the sequence is called RevDR2 (Fig. 2). In comparison to NR1D1 monomer binding to the Rev monomer site, this interaction is 5 to 10 times more stable [2, 16]. In some circumstances, two NR1D1 molecules can individually link to two nearby ROREs and recruit co-repressors (NCOR1-HDAC3) to repress gene transcription [16].

## The role of NR1D1 in the biological clock

**Fig. 3:**
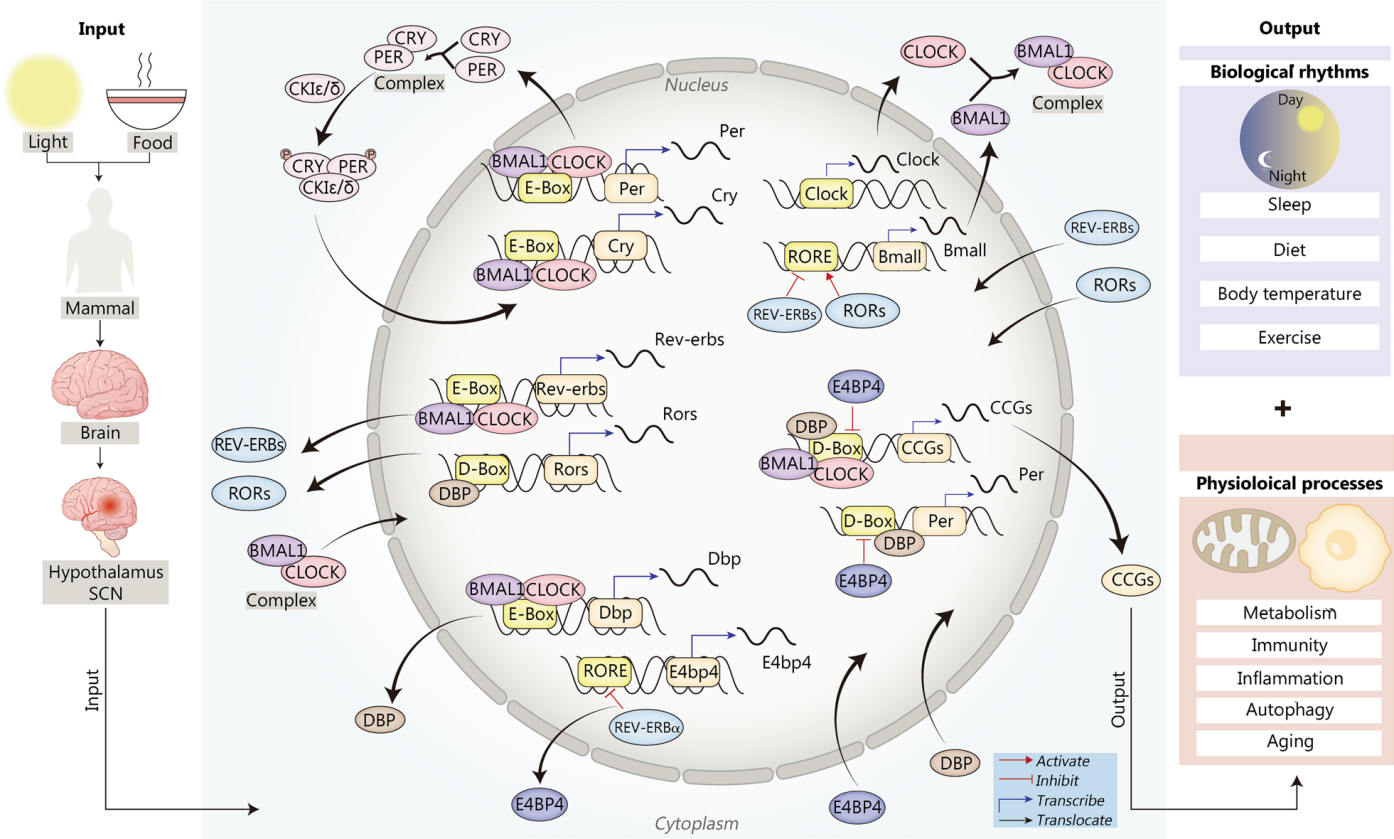
Transcription-translation feedback network of the central circadian oscillator in mammals. Input of light signal and diet signal to the SCN of the mammalian hypothalamus produces transcriptional activation of *Per*, which regulates the PER concentration and subsequently affects biological clock phasing. Mechanically, the core clock proteins BMAL1 and CLOCK form BMAL1-CLOCK heterodimer, translocating into the nucleus and binding to the E-box containing DNA region upstream of the promoters of downstream genes. Then, the transcription of downstream genes is activated, including *Per*, *Cry*, *Rev-erbs* and DBPs and other CCGs. The products of these genes partially translocate back to the nucleus, which feedback regulates the transcription of other clock genes. For example, PER and CRY form a complex that is phosphorylated by CKIε/δ, and subsequently returns to the nucleus to inhibit the activation of the BMAL1/CLOCK complex; REV-ERBs and RORs exert negative and positive regulation of *Bmal1* transcription by competitively binding to the RORE in the promoter, respectively; DBP translocates to the nucleus and activates the transcription of *Rors*, *Per*, and CCGs. Subsequently, the activation of CCGs regulates the output of multiple circadian behaviors, such as biological rhythms and several physiological processes. BMAL1 brain and muscle ARNT-like 1, CCGs clock control genes, CLOCK circadian locomotor output cycles kaput, CRY cryptochrome, CKIε/δ cyclin-dependent kinase inhibitor protein ε/δ, DBP D site binding protein, PER period, ROR RAR-related orphan receptor, E4bp4 E4 promoter binding protein 4, SCN suprachiasmatic nucleus

Since the eighteenth century, research on the mechanism of the circadian clock has been underway. Konopka et al. [18] originally used Drosophila as a model system to investigate biological clock genes, and they discovered clock genes in Drosophila mutants. There is a central “master” clock located in the suprachiasmatic nucleus (SCN) of the mammalian hypothalamus that integrates information from light and synchronizes our physiology to the day/night cycle (Fig. 3). In fact, many rhythmic activities are mediated by peripheral oscillators in various tissues and cells, and the central clock in the brain coordinates various rhythmic activities in different tissues [19].

Circadian rhythms are the biological clock-generated 24-h behavioral and physiological rhythms found in many species. In mammals, the biological clock is housed in the SCN. On the one hand, the master pacemaker in the SCN receives a principal entraining signal from the environmental light-dark cycle [20, 21]. On the other hand, it maintains the synchronized rhythms of behavior and physiology in cells and tissues by aligning circadian gene oscillations within extra-SCN neurons and peripheral tissues [19, 22, 23]. Nearly all physiology is regulated by the circadian clock, and disturbances have serious detrimental effects on health [24]. The most important mechanism by which circadian rhythms can be maintained on an approximately 24 h cycle is the transcription-translation feedback loop (TTFL) of the biological clock. The internal clock consists of many genes and the proteins that they encode, such as brain and muscle ARNT-like 1 (BMAL1), circadian locomotor output cycles kaput (CLOCK), Period (PER), Cryptochrome (CRY), REV-ERBα and RAR-related orphan receptor α (RORα), all of which affect different physiological processes in the body via TTFLs. The central molecular circadian oscillator loop consists of the BMAL1/CLOCK heterodimer. CLOCK and BMAL1 interact with the E-box in the promoter regions of *Per* and *Cry* in mammals, triggering the transcription of these two genes in the nucleus [25]. These genes are subsequently translated into the target proteins PER1-3 and CRY1-2 in the cytoplasm. Conversely, PER1-3 and CRY1-2 can suppress the transcription of CLOCK and BMAL1, forming a negative feedback loop [26]. Importantly, numerous clock-controlled genes (CCGs) are located downstream of these four components and coordinate the oscillation of multiple physiological functions. The nuclear receptors REV-ERBs and RORs form the second feedback loop. Daily oscillations in *Rev-erbs* transcription are caused by CLOCK/BMAL binding to the E-box of the promoter [27]. REV-ERBα is a major repressor of *Bmal1* transcription, and RORα is a transcriptional activator. The two factors compete to bind the RORE/RevRE site located in the *Bmal1* promoter and then regulate the transcription of *Bmal1* and RORE/RevRE-controlled genes (RCGs) [27]. Therefore, REV-ERBα and RORα engage in the regulatory circuit and are essential for the appropriate timing of the core clock mechanism and the occupancy of the *Bmal1* promoter. In the third loop, PER2 (an output gene product from the main loop) and D-box controlled genes (DCGs) are influenced by DBP and E4 promoter binding protein 4 (E4BP4) [28].

Although the patterns differ, all clock genes are expressed cyclically. Notably, numerous clock-controlled genes (CCGs), such as *Bmal1* and *E4bp4*, are under the control of NR1D1 and exhibit distinctive patterns in contrast to NR1D1 [25]. Accordingly, NR1D1, which is a transcription repressor, is one of the crucial players that controls negative feedback mechanisms of the biological clock.

## NR1D1 in autophagy, immunity, inflammation, metabolism and aging

In addition to the regulation in circadian rhythms, NR1D1 also performs as a transcriptional repressor in numerous crucial biological processes, including autophagy, immunity, inflammation, metabolism, and aging.

### NR1D1 in autophagy

Autophagy is a highly conserved intracellular degradation system, and is essential for maintaining cellular homeostasis during stress conditions. NR1D1 participates in autophagy in various organelles, including mitochondria and lysosomes.

Adipocytes, macrophages, and granulosa cells (GCs) are important cell types associated with NR1D1-regulated autophagy. NR1D1 deficiency reduces mitochondrial synthesis and accelerates the clearance of mitochondria in skeletal muscle by increasing mitochondrial autophagy, decreasing mitochondrial quantity, and impairing respiratory chain function [29]. Unc-51-like kinase 1 (ULK1) is a beneficial partner of NR1D1 in mitochondrial autophagy, as determined by Ferder et al. [30]. NR1D1 upregulates ULK1 (essential for autophagy onset) expression in adipocytes by interacting with the *Ulk1* promoter [30]. In addition to ULK1, NR1D1 also regulates autophagy rhythms via other autophagy associated genes. *Nr1d1* knockdown increases autophagy protein 5 (ATG5) expression in mouse GCs. In contrast, rapamycin-induced autophagy and ATG5 expression are partly inhibited by treatment with SR9009 (NR1D1 agonist), indicating that NR1D1 maintains autophagy homeostasis in mouse GCs [31]. Wu et al. [32] discovered that in GSK4112 (an NR1D1 agonist)-treated tilapia, the majority of autophagy-related genes were decreased and exhibited altered rhythmicity involving *Atg4c, Bnip3la, Lc3a, Lc3b*, and *Lc3c* mRNA levels. Moreover, Chandra et al. [33] demonstrated that NR1D1 activation by GSK4112 stimulated an increase in autophagic flux and lysosome formation in human macrophages via transcription factor EB (TFEB)-associated pathways. Molecularly, TFEB and transcription factor E3 (TFE3), which are the main drivers of autophagy and lysosomal biogenesis, directly bind to the *Nr1d1* promoter without the formation of the BMAL1-CLOCK complex to regulate *Nr1d1* expression. This indicates that TFEB and TFE3 collaborate with the fundamental components of the clock mechanism. Endogenous *Nr1d1* knockdown triggers the overexpression of TFEB and TFE3, which leads to enhanced autophagic flux. Thus, the rival but interconnected forces controlled by NR1D1, TFEB and TFE3 determine the duration of autophagy activation to some extent [34].

### NR1D1 in inflammation

NR1D1 plays essential roles in inflammation mediated by multiple cell types, among which macrophages are the primary effector cells of inflammation associated with NR1D1 and the circadian clock. NR1D1 modulates inflammation through various mechanisms, such as reducing the secretion of inflammatory cytokines, regulating gene transcription and the NLRP3 inflammasome pathway, and inhibiting macrophage polarization.

Gibbs et al. [35] demonstrated that administration of a synthetic NR1D1 ligand effectively modulated the generation and secretion of IL-6 (a proinflammatory cytokine), and decreased the mRNA expression of *Tlr4*, *Cxcl11*, *Ccl2*, *Cxcl6*, and *Il19*. GSK4112 or SR9011 (the agonists of NR1D1) dose-dependently suppresses the expression of proinflammatory cytokines [such as interleukin-6 (IL-6) and tumor necrosis factor-α (TNF-α)] through the nuclear factor kappa-B (NF-κB) pathway, thereby mitigating microglia-mediated neuroinflammation [36]. Pharmacological activation of NR1D1 by SR9009 attenuates the release of several inflammatory cytokines, including IL-1β, IL-6, IL-8, IL-18 and TNF-α, and suppresses Toll-like receptor 4 (TLR4)-regulated NF-κB activation and the inflammatory response in human endometrial stroma cells (hESCs) [37].

Lipopolysaccharides (LPS) is not only a typical proinflammatory mediator, but also promotes M1-like macrophage polarization [38]. LPS inhibits the expression of NR1D1 in macrophages, whereas SR9009 inhibits M1 polarization in differentiated macrophages induced by LPS via the phosphatidylinositol-3-kinase (PI3K) signaling pathway [38]. Likewise, NR1D1 activation reduces matrix metalloproteinase (MMP) and chondrocyte levels, and inhibits the polarization of M1 macrophages from fibroblast-like synoviocytes (FLSs) [39–41]. In particular, NR1D1 inhibits the transcription of eRNAs, which are short RNA strands generated from an enhancer site that support enhancer versatility, and inactivates the transcription of adjacent genes (including *Mmp9* and the chemokine receptor *Cx3cr1*) in macrophages [6]. Additionally, Wang et al. [42] found that the NLRP3 inflammasome, which is a key platform that stimulates the maturation and production of proinflammatory cytokines, is inactivated by NR1D1 mainly during the priming stage in mouse primary macrophages. Mechanistically, NR1D1 not only directly inhibits *Nlrp3* transcription by binding to its promoter, but also indirectly suppresses NLRP3 by blocking the NF-κB pathway.

### NR1D1 in immunity

NR1D1 functions as a clock output node, connecting cellular circadian clocks with innate immune responses. Innate immunity is accompanied by inflammation, and inflammatory cells secrete inflammation-related cytokines to participate in immune defense in response to foreign pathogens.

After being activated, naïve CD4 T cells develop into various T helper (Th) subtypes that generate lineage-specific cytokines. Effector Th subtypes play essential roles in coordinating immune responses to a range of infections and are involved in the pathogenesis of numerous inflammatory illnesses, including autoimmunity, allergy, and asthma, by producing unique sets of cytokines. The principal transcription factor for Th2 cell differentiation is GATA binding protein 3 (GATA3) [43]. NR1D1 directly binds to the *Gata3* promoter and interacts with its cellular companion NCOR-HDAC3 to create a durable repression complex, ultimately restricting Th2 cell production [44]. Retinoic acid-related orphan receptor γt (RORγt) is the master transcription factor for the differentiation of interleukin-17-producing CD4 Th17 cells, which are a class of proinflammatory immune cells that protect mucosal surfaces from bacterial and fungal infections [45, 46]. Yu et al. [47] found that E4BP4 inhibited *Rorγt* transcription by binding to GTTACTTAA sequence on the *Rorγt* promoter, thereby restricting Th17 cell differentiation. Moreover, NR1D1 is associated with the development and biological clock of Th17 cells by binding to the consensus sequence of the E4BP4 locus and directly repressing E4BP4 transcription. This guarantees that Th17 lineage specification preferentially emerges at a specific stage of the circadian cycle instead of at random times during the day-night cycle, thus preventing the excessive accumulation of Th17 cells.

Of note, Zhuang et al. [48] highlighted an innovative role for NR1D1 in limiting RNA virus replication, which opens up promising therapeutic possibilities for treating infectious disorders. The *Flaviviridae* family of positive-strand RNA viruses is the major pathogen in several diseases with high morbidity and mortality and includes the human pathogens hepatitis C virus (HCV), dengue virus (DENV) and Zika virus (ZIKV). Pharmacological activation of NR1D1 prevents HCV entry and restricts the replication of HCV, DENV and ZIKV RNA by disrupting fatty acid metabolism and stearoyl-CoA-desaturase (SCD) activity [48]. However, a recent study revealed that NR1D1 could also impair the host defense response. In gastric epithelial cells (GECs) infected with *Helicobacter pylori*, NR1D1 not only directly suppresses the expression of antibacterial proteins (including Reg3b and β-defensin-1) to impaire bactericidal effects against *Helicobacter pylori*, but also directly limits production of the chemokine (C-C motif) ligand 21 (CCL21) as a consequence of the diminished bacterial clearance capacity of the *Helicobacter pylori*-specific Th1 cell response.

### NR1D1 in metabolism

Numerous studies have shown that NR1D1 is a crucial and novel physiological regulator of lipid metabolism, glucose metabolism and insulin resistance.

Raspé et al. [49] found that apolipoprotein (apo)C-III expression was positively correlated with the risk of cardiovascular disease development and identified NR1D1 as a biological repressor of apoC-III gene transcription. The expression of rat apo A-I` [a crucial component of high-density lipoproteins (HDL)] and apoC-III (an apolipoprotein implicated in the metabolism of triglyceride-rich lipoproteins) are suppressed by NR1D1 through binding to the AGGTCA half-site located in the apoA-I or apoC-III promoter [49, 50]. Notably, NR1D1 inhibits transcription of both apolipoproteins, whereas RORα activates apoA-I and apoC-III transcription after binding to the same response element, demonstrating the cross-talk between these nuclear receptors and common target genes. In addition, NR1D1 participates in the transcriptional regulation of various lipid metabolism-related enzymes. *Elovl3*, a gene that codes for an extremely long-chain fatty acid elongase, is suppressed by NR1D1 [51]. NR1D1 also modulates peroxisome proliferators-activated receptor α (PPARα)/retinoid X receptor α (RXRα)-dependent transactivation in a response element-specific manner and reduces the expression of enoyl CoA hydratase/3-hydroxyacyl CoA dehydrogenase, which participates in the peroxisomal β-oxidation pathway [52]. Cytochrome P450 (CYP450), which are a superfamily of enzymes containing heme as a cofactor, play important roles in the clearance of many substances (such as oxidized steroids, fatty acids, and xenobiotics) and the synthesis and catabolism of hormones in mammals. An alternative pathway for fatty acid metabolism is lipid ω-hydroxylation of medium- and long-chain fatty acids metabolized by the cytochrome CYP4A family. According to Yang et al. [53], NR1D1 inhibits the transcription of *Cyp4a10* and *Cyp4a14*, and NR1D1 deficiency significantly increases the expression levels of both factors, which promotes lipid accumulation and oxidative stress. Additionally, NR1D1 enhances serum cholesterol levels and hepatic cholesterol accumulation by inhibiting the production of cholesterol 7α-hydroxylase expression (CYP7A1), an enzyme that converts cholesterol into bile acids.

Various glucose metabolic pathways, including gluconeogenesis, the pentose phosphate pathway (PPP), glycolysis, and the tricarboxylic acid (TCA) cycle, are also directly and indirectly influenced by NR1D1. NR1D1 was identified as a putative apoA-IV-binding protein by Li et al. [54]. In 2013, researchers found that apoA-IV could bind to and activate NR1D1 to suppress the expression of phosphoenolpyruvate carboxykinase (PEPCK) and glucose-6-phosphatase (G6Pase) in hepatocytes and decrease hepatic glucose production [54]. In 2015, they showed that apoA-IV and nuclear receptor subfamily 4, group A, member 1 (NR4A1) interacts at the RORα response element in the human G6Pase promoter, and both factors could mediate transcriptional repression of the *G6Pase* and *Pepck* genes by connecting with NR1D1 to reduce hepatic glucose output and lower blood glucose [55]. Increased NR4A1 expression induced by apoA-IV in hepatocytes further inhibited gluconeogenesis, and NR1D1 and NR4A1 could serve similar or complementary roles in the apoA-IV-mediated regulation of gluconeogenesis. During gluconeogenesis, phosphoenolpyruvate carboxykinase 1 (PCK1) is the rate-limiting enzyme. SR9009 treatment of human HepG2 hepatoma cells significantly lowers PCK1 expression by directly binding to the − 325 to − 320 bp region (a RevRE site) in the gene promoter to lower plasma glucose [56]. Similarly, the genes expression of hexokinase II, transketolase, and ribose-5-phosphate isomerase are elevated by NR1D1 deletion, thereby affecting glucose metabolism. Finally, NR1D1 is also involved in insulin resistance. Fibroblast growth factor-21 (FGF21) is a hepatic hormone that potently improves peripheral insulin sensitivity and lipid metabolism. NR1D1 binds to the RORE sites of FGF21 and negatively regulates FGF21 expression, thereby inhibiting EGF21 to improve insulin sensitivity [57].

### NR1D1 in aging

Aging is a key factor in the development of multiple diseases, and it has been reported that the circadian rhythms of many organs/cells are disrupted with age. Recently, many studies have also focused on the potential of NR1D1 as a therapeutic target for aging-related diseases.

Retinal epithelial function declines with age, mainly due to the accumulation of oxidative stress. Huang et al. [58] performed pharmacological activation of NR1D1 and found that the monomer could directly bind to RORE/RevRE (in a non-NCOR1/HDAC3-dependent manner) to modulate the transcription of nuclear factor erythroid 2-related factor 2 (NRF2) and its downstream antioxidant enzymes superoxide dismutase 1 (SOD1) and catalase, thereby attenuating retinal pigment epithelial and retinal damage and ameliorating oxidative stress in mice with age-related macular degeneration (AMD). Notably, NR1D1 levels were different in young and aged heart-derived Sca-1^+^ CD31^−^ cells, which are resident cardiac progenitor cells that can differentiate into cardiomyocytes. In young heart-derived Sca-1 CD31 cells, higher levels of NR1D1 inhibit cell proliferation and promote apoptosis. Conversely, downregulation of NR1D1 in the latter promotes cell proliferation and inhibits apoptosis by blocking the G_0_/G_1_ phase of the cell cycle. Furthermore, Pu et al. [59] demonstrated that NR1D1 inhibits the expression of NR3A4 by binding to its promoter, which allows NR3A4 to further interact with the promoter of serine protease inhibitory factor 3 (*Serpina3*, a gene associated with apoptosis inhibition), ultimately attenuating the transcriptional repression of *Serpina3* and exerting an antiapoptotic effect. Interestingly, NR1D1 is sex-differentiated during liver aging, and its expression is higher in the aging livers of male rats than in those of female rats, while the opposite is true for the expression of many its downstream circadian genes [60].

## Physiological and pathological roles of NR1D1 in various organs

**Fig. 4:**
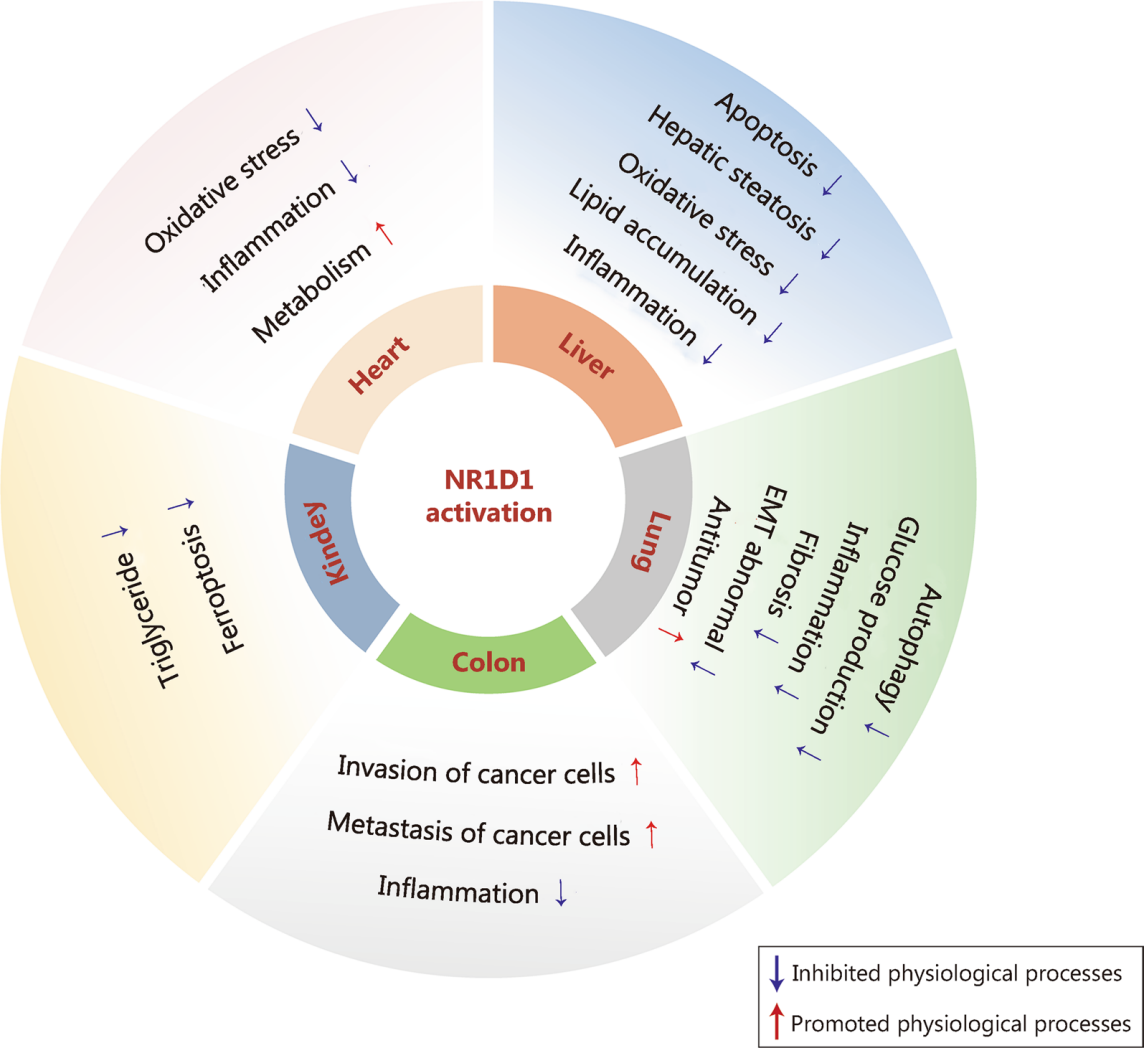
Regulation of multiple physiological processes by NR1D1 in various organs. An overview of the effects of NR1D1 on autophagy, inflammation, metabolism, oxidative stress, apoptosis and other physiological processes in various organs. NR1D1 nuclear receptor subfamily 1, group D, member 1

NR1D1 is widely expressed in numerous tissues or organs and exerts many biological effects on heart, liver, lung, kidney and many other organ injuries (Table 1; Fig. 4).

**Table 1:** Pathophysiological effects of NR1D1 in different organs

Organ	Disease	Models/Materials	Effects	References
Heart	MI	*Nr1d1*^−/−^ mice	SR9009 lowers NLRP3 inflammasome level in myocardial fibroblasts and immunocyte recruitment to heal the vulnerable infarct	[61]
	HF	MI mouse model induced by the permanent ligation of the left LAD	SR9009 reduces adverse cardiac remodeling through alleviating inflammation	[62]
		Pressure overload mouse model caused by the constriction of aortic arch between the right and the left carotid arteries	NR1D1 represses the transcription of numerous genes involved in cardiomyocyte hypertrophy in vitro. SR9009 enhances fatty acid oxidation via increasing PDK4 expression, and blocks the cellular remodeling induced by pressure overload in vivo	[8]
	Thrombus	BMDMs and *NR1D1*^*−/−*^ mouse model of rupture-prone vulnerable plaques induced by partly ligating the left renal artery and the left internal and the external carotid arteries	NR1D1 activation inhibits macrophage pyroptosis in a NF-κB/NLRP3 inflammasome-dependent manner in BMDMs, and mitigates macrophage infiltration, inflammation, and oxidative stress to stabilize rupture-prone vulnerable plaques	[63]
		Acute MI mouse model induced by ligation of LAD	NR1D1 potentiate platelet aggregation and activation via the OPHN-1/RhoA/ERM signaling mediated by oligophrenin-1	[64]
Liver	FH	Naïve peritoneal macrophages and primary BMDMs from *Nr1d1*^*−/−*^ mice and *Nr1d1*^*+/+*^ littermates	NR1D1 inhibits the NLRP3 inflammasome signaling, reduces inflammatory cytokines and CCL2-mediated hepatic infiltration of innate immune cells	[7]
	ALD	*Shp*^*−/−*^ mice fed modified ethanol-binge	NR1D1 reduces lipid accumulation and oxidative stress via SHP / REV-ERBα / CYP4A axis	[53]
	NAFLD	Whole-body or hepatocyte-specific mPGES-2-deficient mice fed a high-fat or methionine-choline-deficient diet	NR1D1 decreases CYP4A14 and increases acyl-CoA thioesterase 4 levels to potentiate lipid metabolism	[65]
	NAFLD	*Nr1d1* Δex3/4 mice fed high fat diet	The deletion of exons 3 and 4 in the mouse *Nr1d1* gene worsens HFD-induced hepatic steatosis	[66]
	Apoptotic liver injury	Acute hepatic damage mouse model induced by Fas	GSK4112 decreases the level of Fas and the activity of caspase-3 and caspase-8, suppressing hepatocyte apoptosis	[67]
Lung	ALI	ALI mouse model made by intraperitoneal injection of LPS	NR1D1 reduces lung vascular permeability and inflammatory cells infiltration via inhibiting the NF-κB /NLRP3 pathway	[9]
	Cigarette smoke -induced lung inflammation	Lung inflammation mouse model induced by cigarette smoke	GSK4112 decreases the release of inflammatory cytokines	[68]
	Lung adenocarcinoma	Lung adenocarcinoma cell line A549	Downregulation of NR1D1 stimulates the invasion and promotes the proliferation of lung adenocarcinoma cell line A549	[69]
	Lung adenocarcinoma	Nontumorigenic mouse hepatocyte cell line AML12	NR1D1 is targeted and destabilized by PKA, resulting in increased glucose production	[70]
	SCLC	Chemosensitive SCLC cells (H69 and H446) and the corresponding chemoresistant SCLC cells (H69AR and H446DDP)	SR9009 directly represses the autophagy gene *Atg5* to suppress SCLC cell autophagy activity	[71]
Kidney	AKI	*NR1D1*^−/−^ mice and AKI mouse model induced by folic acid and AAI	NR1D1 represses the transcription of *Slc7a11* and *HO1* to promote ferroptosis, and loss of NR1D1 reduces the sensitivity of mice to AKI and eliminates the circadian time dependency in disease severity	[72, 73]
Colon	Colitis	BMDMs and colitis mouse model induced by dextran sulfate sodium salt	NR1D1 plays an anti-inflammatory role and affects the circadian rhythm of colitis through direct activation by berberine	[74]

*AAI* aristolochic acid I, *AKI* acute kidney injury, *Atg5* Autophagy protein 5, *BMDMs* bone marrow-derived macrophages, *ERM* ezrin/radixin/moesin, *HF* heart failure, *HO1* heme oxygenase, *LAD* left anterior descending coronary artery, *LPS* lipopolysaccharide, *MI* myocardial infarction, *mPGES-2* microsomal prostaglandin E synthase-2, *NLRP3* NOD-like receptor thermal protein domain associated protein 3, *NF-κB* nuclear factor kappa-B, *NR1D1* nuclear receptor subfamily 1, group D, member 1, *OPHN-1* oligophrenin 1, *PDK4* pyruvate dehydrogenase kinase 4, *PKA* protein kinase A, *RhoA* Ras homolog gene family, member A, *SCLC* small-cell lung cancer, *Slc7a11* solute carrier family 7 member 11, *SHP* small heterodimer partner

### NR1D1 in the heart

The heart provides sufficient blood flow, oxygen, and various nutrients to organs and tissues and removes the end products of metabolism to maintain normal cellular metabolism and function.

Myocardial infarction (MI) is the blockage of the coronary arteries of the heart, resulting in massive ischemic necrosis in myocardial cells and serious complications. Reperfusion may trigger cardiac inflammation, infarct expansion, and heart failure (HF) after MI. Reitz et al. [61] identified cardiac fibroblasts as the target cells of SR9009 and showed that SR9009 inhibited the production of the NLRP3 inflammasome and the recruitment of immune cells to the heart, thereby healing vulnerable infarcts, improving adverse cardiac remodeling and facilitating long-term cardiac repair and reperfusion in MI mice [61, 62]. Furthermore, Zhang et al. [8] demonstrated that SR9009 enhanced several metabolic molecules and pathways that were downregulated in HF mice, especially pyruvate dehydrogenase kinase 4 (PDK4), which is an important regulator of fatty acid oxidation. The researchers observed that PDK4 expression increased when NR1D1 was linked to the *Pdk4* enhancer, suggesting that PDK4 was one of the principal targets of NR1D1 during cardiac metabolic remodeling [8].

NR1D1 ameliorates thrombosis-based cardiovascular disorders associated with day/night cycles in animal models. The major cause of acute cardiovascular events is thrombosis, which can result from vulnerable plaque rupture. NR1D1 plays a protective role in the vasculature by modulating inflammation and oxidative stress to stabilize fragile plaques. According to Wu et al. [63], NR1D1 deficiency increased macrophage infiltration, inflammation, and oxidative stress, which enhanced the fragility of the plaque and could cause it to spontaneously rupture with intraluminal thrombosis. In mouse bone marrow-derived macrophages (BMDMs), NR1D1 activation reduces macrophage pyroptosis via the NF-κB/NLRP3 inflammasome pathway, and decreases plaque susceptibility and rupture [63]. Notably, NR1D1 is expressed in platelets and functions as a positive thrombosis regulator, potentiating platelet activation and aggregation via oligophrenin-1-mediated OPHN-1/RhoA/ERM signaling [64]. Moreover, NR1D1 decreases ferric chloride-induced carotid artery occlusive thrombosis and protects against microvascular microthrombi blockade and infarct growth in an acute MI model [64].

Taken together, these studies may deepen our comprehension of NR1D1 in many physiological processes and emphasize the importance of circadian clock mechanisms in platelet physiology and MI, paving the way for further studies on NR1D1-targeted therapeutics.

### NR1D1 in the liver

The liver is referred to as the center of substance metabolism, and is crucial to all bodily functions, including digestion, absorption, excretion, biotransformation, and metabolism. Acute liver injury caused by hemorrhagic necrosis, extensive hepatocyte apoptosis and inflammation is known as fulminant hepatitis (FH). LPS/D-galactosamine (GalN)-induced FH mice with NR1D1 activation exhibits reduced CCL2-mediated hepatic infiltration of innate immune cells and inhibits activation of the NLRP3 inflammasome pathway, and these mice have higher survival rates [7]. On the one hand, SR9009 inhibits the recruitment of infiltrating monocytes, macrophages and neutrophils by preventing the increase in hepatic F4/80 and CCL2 expression. On the other hand, treatment with SR9009 lowers the expression of NLRP3, IL-1β and IL-18 in macrophages and subsequently attenuates NLRP3-driven inflammation [7].

One of the liver’s early detoxifying reactions to excessive alcohol consumption is hepatocyte lipid production, which ultimately results in alcoholic liver disease (ALD). Hepatocytes undergo steatosis when lipid ω-hydroxylation is inhibited. NR1D1 is a potential circadian transcriptional repressor of murine *Cyp4a10* and *Cyp4a14*, which are extensively expressed in the liver and are similar to human CYP4A22 and CYP4A11 respectively [53]. Mechanistically, the DNA-binding structural domain of NR1D1 binds to the *Cyp4a10* and *Cyp4a14* promoters in the mouse liver, inhibiting their activation to reduce lipid accumulation and oxidative stress in hepatocytes [53]. Yang et al. [53, 75] also reported that the lack of small heterodimer partner (SHP) attenuated the ethanol-induced lipid hydroxylation pathway, while its overexpression reduced the repressive effect of NR1D1 on both promoters. Hence, NR1D1 is a potential therapeutic target for ALD via the SHP/NR1D1/CYP4A axis. Additionally, NR1D1 improves nonalcoholic fatty liver disease (NAFLD) and reduces alcohol-induced hepatic steatosis. Zhong et al. [65] demonstrated that NR1D1 protected against NAFLD by decreasing CYP4A14 and increasing acyl-CoA thioesterase 4 levels in microsomal prostaglandin E synthase-2 (mPGES-2)-deficient mice. Genetically, the deletion of exons 3 and 4 in the mouse *Nr1d1* gene worsens high-fat diet-induced hepatic steatosis, according to Hyelin et al. [66]. Therefore, NR1D1 is a key regulator in both high-fat and alcohol-induced hepatic steatosis.

An important mechanism of hepatocyte damage during acute and chronic hepatic diseases, such as virus-driven hepatitis, ALD, NAFLD, and ischemia/reperfusion (I/R)-induced liver injury, is the aberrant activation of apoptosis by Fas (a death receptor also known as CD95). Fortunately, treatment with GSK4112 improves liver damage caused by Fas in mice [67]. Mechanistically, GSK4112 reduces Fas levels, enhances Akt phosphorylation, and lowers caspase-3 and caspase-8 activity, thereby inhibiting hepatocyte apoptosis and ameliorating liver injury. In summary, NR1D1 agonists may be helpful for the pharmacological treatment of liver damage caused by Fas.

### NR1D1 in the lung

As the most important part of the respiratory system, the lung delivers oxygen to the capillaries and expels carbon dioxide from the blood to sustain human life, in addition to its role in immunity, defense, metabolism, and blood storage. Pulmonary inflammation is the most common infectious disease of the respiratory system and can develop into chronic obstructive pulmonary disease (COPD) or even carcinoma of the lung.

NR1D1 ameliorates LPS-induced acute lung injury (ALI) [9]. On the one hand, NR1D1 inhibits the DNA binding activity of NF-κB and blocks p65 nuclear activation induced by LPS in RAW 264.7 macrophages [9]. On the other hand, NR1D1 dramatically downregulates the NLRP3 inflammasome, ASC and caspase-1, which reduces IL-1β production in RAW 264.7 macrophages [9]. In addition, NR1D1 activation significantly attenuates cigarette smoke (CS)-induced lung inflammatory responses, while *NR1D1* knockdown exacerbates circadian rhythm disruption and epithelial-mesenchymal transition (EMT) dysregulation in the lungs of mice with chronic CS exposure [68]. In vitro, GSK4112 pretreatment inhibits transforming growth factor (TGF)- and cigarette smoke-induced fibroblast differentiation in human fetal lung fibroblast 1 (HFL-1) calls and reduces CS extract/LPS-induced pro-inflammatory cytokine release from primary human small airway epithelial cells (SAECs) and mouse lung fibroblasts (MLFs) [68, 76]. In addition to improving inflammation, treatment with SR9009 reduces acute CS-induced aberrant EMT in the lung [76]. Lung carcinoma is one of the most common cancers, and its incidence and mortality have been increasing for half a century. The histological manifestations of lung cancer are complex and diverse, including lung adenocarcinoma and small-cell lung cancer (SCLC). Downregulation of NR1D1 significantly enhances NF-κB transcription, stimulates invasion and promotes proliferation of the lung adenocarcinoma cell line A549 [69]. Moreover, hepatic protein kinase A (PKA) signaling is activated by an increase in glucagon and destabilizes NR1D1, resulting in increased glucose production in a lung adenocarcinoma cachexia model [70]. In subcutaneous tumor models of SCLC, the NR1D1 agonist SR9009 has been shown to have antitumor effects [71]. After NR1D1 activation by SR9009, NR1D1 directly binds to the *Atg5* (a key autophagy gene) promoter and inhibits its activity, thereby inhibiting autophagic activity and inducing SCLC cell-specific toxicity. In addition, SR9009 activates the apoptosis proteins poly-ADP-ribose polymerase (PARP) and caspase 3 to induce apoptosis and exert antitumor effects [71]. Elucidating the relationship between circadian clock components and autophagic activity may contribute to the discovery of new therapeutic targets for SCLC. Pharmacological modulation of biological clock components via SR9009 is a novel and promising therapeutic approach for SCLC.

### NR1D1 in the kidney

The kidney is responsible for filtering metabolic waste and excreting it from the body and reabsorbing different nutrients into the blood circulation system. Mészáros et al. [77] explored the ontogeny of the clock system in the kidney. The researchers found that the kidney governed the circadian rhythm of fluid and electrolyte excretion, which was important for maintaining homeostasis. Renal cell carcinoma (RCC) is a frequent malignant tumor with a high prevalence and a dismal prognosis. A study of RCC prognosis showed that in the kidney tissue of mice, 13 rhythmic genes varied in circadian rhythm, and there was increased NR1D1 expression and methylation levels in cancer cells [10]. This finding demonstrated that biological clock rhythms have a significant influence on RCC and offer a solid foundation for future RCC diagnosis, prognosis and medication recommendations. In addition, AKI is a prevalent and serious illness that has severe morbidity and high mortality, and NR1D1 is a crucial promotor of acute renal injury (AKI) induced by folic acid [72]. When NR1D1 is knocked out, the sensitivity of mice to folic acid-induced AKI is decreased, and the circadian clock dependency of disease severity is removed. The key factor in folic acid-induced AKI is ferroptosis. By directly binding to a RORE cis-element, NR1D1 suppresses the transcription of Slc7a11 and HO1, two ferroptosis-inhibitory genes, thereby increasing folic acid-induced AKI [72]. Similarly, NR1D1 inhibition ameliorates aristolochic acid I (AAI)-induced kidney injury in mice by limiting ferroptosis [73]. Therefore, these results may have repercussions for our comprehension of circadian clock-controlled kidney physiopathology and the development of novel treatments for kidney injury.

### NR1D1 in other organs

As discussed previously, NR1D1 participates in physiological and pathological processes in the heart, liver, lung, and kidney, and it may play essential roles in other organs/tissues. The anti-inflammatory effect of berberine is NR1D1-dependent. Berberine alleviates colitis in vivo and in vitro, but this effect is abolished in BMDMs from NR1D1-deficient mice [74]. Mechanistically, berberine attenuates the inflammatory response to colitis in BMDMs and colitis mice by activating the NR1D1/AMPK pathway and inhibiting the activator protein 1 (AP-1) and NF-κB pathways [74]. These findings could affect how chronotherapy is used to treat colitis or other similar disorders.

In vertebrates, stimulated by retinoic acid gene 8 (*Stra8*) is a crucial gatekeeper for meiotic initiation. Meiosis failure and autophagy activation in *Stra8*-deficient germ cells suggest that STRA8 may inhibit autophagy during the formation of germ cells in sexual organs [30]. NR1D1 has been shown to be a direct target of *Stra8* transcriptional suppression by Ferder et al. [30]. The promoter of *Ulk1*, a gene that is required for the initiation of autophagy, is also bound by NR1D1 in *Stra8*-deficient testes, and NR1D1 is required for the increased expression of Ulk1 [30]. It was suggested that STRA8 reduces germ cell autophagy by directly inhibiting NR1D1, which is required for the production of the crucial autophagy initiator ULK1 [30]. The STRA8/NR1D1/ULK1 axis thus proposes a unique relationship between autophagy suppression and meiotic initiation of testis germ cells.

Skin inflammation is a key causative agent of several skin-related diseases, including acne and psoriasis. NR1D1 improves skin inflammation induced by *Propionibacterium acnes* by inhibiting *Bmal1* transcription and the downstream NF-κB/NLRP3 axis, thereby preventing acne [78]. Psoriasis, which is one of the most common inflammatory diseases, is associated with an inflammatory response mediated by Th cells and involves multiple inflammatory cytokines, among which IL-17 plays an important role. Wang et al. [79] found that NR1D1 activation attenuated imiquimod (IMQ)-induced psoriasis-like dermatitis in mice by negatively regulating the secretion of IL-17 by Th17 cells and γδ T cells. Currently, circadian skincare is in the developmental stage and may become a new trend in skincare in the future.

## Ligands of NR1D1

**Fig. 5:**
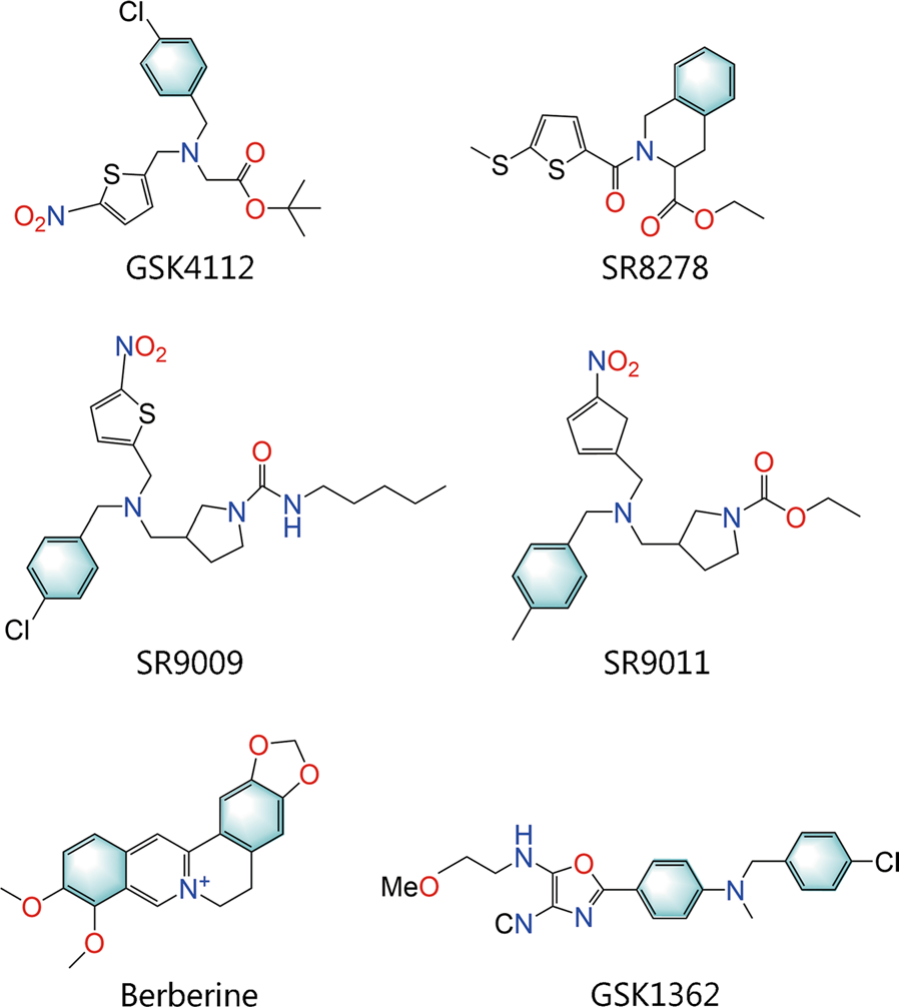
Chemical structures of important NR1D1 ligands. NR1D1 nuclear receptor subfamily 1, group D, member 1

NR1D1 is involved in controlling several pathophysiological processes in numerous organs, including immunological function, metabolism, and circadian rhythm, and is considered as a viable therapeutic target for the treatment of many disorders listed previously. NR1D1 used to be thought of an orphan nuclear receptor, a nuclear receptor without ligand that controls gene transcription in a monomeric or multimeric state. However, a series of NR1D1 ligands have been discovered or synthesized, and NR1D1 is classified as a ligand-dependent receptor. By binding to the receptor’s LBD and causing a conformational shift to enable recognition of a particular motif present in the coactivator protein, ligands control NR-mediated recruitment of coactivators. Fortunately, most ligands of NR1D1 possess agonistic or antagonistic pharmacological activity in vivo and have been validated by several preclinical trials to have pharmacological effects on many diseases or pathological processes. Unfortunately, little progress has been made in the clinical translation of NR1D1 ligands. We will examine the major ligands of NR1D1 and describe them below (Fig. 5; Table 2).

**Table 2:** The overview of NR1D1 ligands

Category	Ligands	Agonist or antagonist	Discoverer	Discovered year	References
Endogenous	Heme	Agonist	Srilatha Raghuram	2007	[80]
Synthetic	GSK4112	Agonist	Qing-Jun Meng	2008	[81]
Synthetic	SR8278	Antagonist	Douglas Kojetin	2011	[82]
Synthetic	SR9009 SR9011	Agonist	Laura A Solt	2012	[83]
Synthetic	THIQ1-4	Agonist	Romain Noel	2012	[84]
Synthetic	GSK2945 GSK0999 GSK5072 GSK2667	Agonist	Ryan P Trump	2013	[85]
Synthetic	SR12418	Agonist	Christina Chang	2019	[86]
Natural	Berberine	Agonist	Zi-Yue Zhou	2020	[74]
Natural	Puerarin	Antagonist	Min Chen	2020	[87]

*NR1D1* nuclear receptor subfamily 1, group D, member 1, *THIQ* tetrahydroisoquinoline

### Natural agonists of NR1D1

Heme is a porphyrin that contains iron and functions as a prosthetic group for oxidative metabolism-related enzymes. In 2005, research on ecdysone-induced protein 75 (E75, the NR1D1 homolog in *Drosophila*) provided clues to a physiological ligand for NR1D1. This finding has demonstrated the need for heme as a ligand and possible binding within the LBD ligand pocket [88]. In 2007, Raghuram et al. [80] successfully identified heme as a ligand for NR1D1 and elucidated its structural basis and physiological mechanisms. Strong transcriptional repression caused by binding between the Fe (II) center and NR1D1 is mainly mediated by His602 and Cys418 (two residues in the C-terminal LBD structural domain of NR1D1), and this binding can be reversed by nitric oxide (NO) [89]. Notably, to strengthen transcriptional repression, this interaction can also increase the recruitment of corepressor proteins such as NCOR [89]. As a heme sensor, NR1D1 helps to maintain heme homeostasis while regulating metabolic and circadian activities, which may be affected by ambient heme concentrations. Wu et al. [90] demonstrated that NR1D1 reduced heme levels by directly repressing *PGC-1* transcription, and heme regulated its own synthesis by stimulating NR1D1-mediated repression of *PGC-1α* through negative feedback. Furthermore, stable expression of NR1D1 leads to intracellular heme deficiency, which reduces the activity of mitochondrial complex iv, thereby inhibiting the respiration-driven oxygen consumption rate and mitochondrial gene expression [90]. Heme binds and activates NR1D1 to regulate the levels of CYP4A14 and acyl-CoA thioesterase 4, thereby contributing to the beneficial effects of mPGES-2 deficiency on NAFLD mice [65, 91]. Thus, NR1D1 may be a therapeutic target in diseases with abnormal heme levels.

Berberine is an isoquinoline alkaloid isolated from the Chinese herb *Coptis chinensis* and other Berberis plants. Zhou et al. [74] examined mice with colitis induced by dextran sulfate sodium in drinking water and demonstrated that berberine reduced myeloperoxidase and malondialdehyde activity, as well as the levels of inflammatory cytokines (IL-1, IL-6, IL-18, and CCL2). The in vitro inflammatory effect of berberine is lost in BMDMs from NR1D1-deficient mice. Moreover, berberine, which is an agonist of NR1D1, significantly enhances the transcriptional repressor activity of NR1D1 and decreases BMAL1 and NLRP3 expression in a dose-dependent manner [74]. It is worth noting that berberine can mediate metabolic regulation and improvements in insulin resistance and exhibit antioxidant, antibacterial, anti-inflammatory, anticancer and other pharmacological effects [92–95]. Berberine also has very low toxicity at the typical dose and exhibits clinical benefits without major side effects. However, due to a lack of research on berberine as an NR1D1 agonist, more efforts and attention are needed. Additionally, the correlation of quercetin, caffeic acid and resveratrol with NR1D1 has been reported, suggesting a wide selection of NR1D1 agonists in nature [96].

### Synthetic agonists of NR1D1

GSK4112 {[1,1-dimethylethyl N-[(4-chlorophenyl) methyl]-N-[(5-nitro-2-thienyl) methyl]}, which is also known as SR6452 and was described by Meng et al. [81], is the first synthetic NR1D1-targeting ligand. Recently, many preclinical studies have confirmed that GSK4112 has multiple pharmacological effects, but its clinical translation has not been demonstrated [97–99]. GSK4112 can be used as an antidiabetic agent because it lowers glucose production in primary hepatocytes [99, 100]. GSK4112 is a potent adipogenesis modulator that suppresses preadipocyte proliferation and promotes apoptosis, suggesting that it may be a biological target for the prevention and treatment of obesity-related diseases [97]. In addition, intraperitoneal administration of GSK4112 protects bone loss by inhibiting osteoclast differentiation in vivo, demonstrating that GSK4112 is a potential therapeutic agent for the treatment of bone disease characterized by excessive bone resorption [98]. Notably, GSK4112 may be an anti-infective compound that protects against intracellular pathogen infection, especially in the context of tuberculosis treatment [33]. In addition to the aforementioned effects of GSK4112 on diabetes, obesity, bone loss, and tuberculosis, GSK4112 has also shown nonnegligible therapeutic potential in inflammation-related lung injury and neuropathic pain [76, 101, 102]. Kojetin et al. [82] discovered that GSK4112 had a poor pharmacokinetic profile after intraperitoneal administration (low systemic exposure) and exhibited limited NR1D1 agonism, thereby restricting its use as a tool to examine NR1D1 function. Given these limitations, further efforts were made by Trump et al. [85] to optimize a series of NR1D1 agonists through three amine substituents, with a focus on improving compound pharmacokinetics and NR selectivity. For example, GSK2945, GSK0999, GSK5072 and GSK2667 exhibit enhanced activity compared to GSK4112, such as significant suppression of BMAL1, decreased activity against liver X receptor α (LXRα), and more than 100-fold selectivity for NR1D1 [85].

Fortunately, Kojetin et al. [5] collaborated with other groups and identified powerful and effective NR1D1 modulators based on the GSK4112 scaffold, providing key compounds for further guidance and in-depth research on NR1D1 function in vitro and in vivo. SR9009 and SR9011 are typical representatives that were first synthesized and functionally tested in 2012 that have a three- to fourfold efficiency compared to GSK4112 [83], and have been extensively shown to pharmacologically target NR1D1. SR9009 and SR9011 undoubtedly have anti-inflammatory effects on various organs and tissues, such as the lung, retina, liver, bone and hippocampus [7, 41, 76, 103, 104]. Moreover, SR9009 and SR9011 exert powerful metabolic effects induced by NR1D1 on processes such as glucose metabolism, lipid metabolism and cholesterol and bile acid metabolism [83]. Solt et al. [83]. showed that SR9009 and SR9011 affected the circadian expression of several core clock genes in the mouse hypothalamus and dose-dependently suppressed the expression of responsive genes in the mouse liver by modulating NR1D1 activity. For example, the suppression of *Srebf1, Scd1, Srebf2* and cholesterol 7α-hydroxylase expression (*Cyp7a1*), an increase in hexokinase 1 *(HK1)* and pyruvate kinase M2 *(Pkm2)*, and a phase shift in the expression of *Fasn* were observed after SR9011 treatment [83]. The strong potency of SR9009 and SR9011 in metabolic processes indicates that these agents could modulate obesity. In fact, SR9009 and SR9011 have been shown to increase energy expenditure without altering exercise behavior or food intake, inducing weight loss in diet-induced obese mice and suggesting a novel approach to weight loss [83]. Additionally, the fundamental clock mechanism is tightly linked to metabolic control, and several examples of genetic changes in clock genes that cause metabolic abnormalities and even metabolic illnesses in mouse models exist [105, 106]. Hence, SR9009 and SR9011 may adversely affect the biological clock of dieters by regulating circadian rhythms. Additionally, SR9009 and SR9011 have been identified as novel antitumor agents that modulate circadian regulators. Both factors have specific lethal effects on cancer cells and oncogene-induced senescent cells, such as breast cancer cells, SCLC cells and melanocytic naevi, but have no influence on normal cell survival [71, 107, 108]. Moreover, according to Dierickx et al. [109], SR9009 has NR1D1-independent effects on the survival and proliferation of hepatocytes and embryonic stem cells, which has an impact on metabolism, gene expression, and mitochondrial respiration. Therefore, SR9009 cannot be used only as a surrogate for the NR1D1 effect. More attention should be focused on its independent activity, and even its conformational resolution can help to develop NR1D1-independent active molecules in the future. Despite this early promise, 10 µmol/L SR9009, SR9011 and GSK4112 have been reported to exhibit off-target binding to LXRα [85], resulting in the limitations of using SR9009, SR9011 and GSK4112 to explore the pharmacological mechanisms of NR1D1. In an attempt to further optimize these compounds, Amir et al. [86, 110] identified the novel NR1D1 agonist SR12418, which was modified from SR9009 with minimal off-target activity and significantly improved plasma exposure in mice. SR12418 inhibits Th17-driven autoimmunity in vivo, suggesting further efficacy in autoimmune diseases.

Several other NR1D1 agonists have also been reported, such as the tetrahydroisoquinoline (THIQ) compounds optimized from the GSK4112 scaffold [84], as well as several 6-substituted triazolopyridazines (including Cpd-4-3 and Cpd-4-99) [WO2013045519A1]. Unfortunately, the pharmacological properties of these agonists have been poorly reported, and their clinical translational potential has not yet been demonstrated. Notably, bias agonists have received increasing attention in recent years. Compared with general ligands, bias agonists differ in their ability to activate two or more downstream signals and modulate specific signaling pathways and have great therapeutic potential. For instance, SR9009 has biased activation properties by activating the PI3K pathway rather than the more general NF-κB signaling pathway to improve M1-like polarization and attenuate inflammation in the LPS-induced decidual macrophages (dMφs) of pregnant mice [38]. These findings suggest that NR1D1 may have different conformations during ligand binding, which allows biased signaling. Therefore, future studies on the development of NR1D1 ligands may take biased agonists as a possible direction to target a signaling pathway more specifically and reduce possible side effects. Moreover, exploring more efficient and specific ligand structural backbones other than the aforementioned GSK series and THIQ scaffolds will also provide further ideas for NR1D1-targeted drug development.

### Natural antagonists of NR1D1

Recently, Chen et al. [87] first identified puerarin, which is isolated from Puerariae radix, as an antagonist of NR1D1. In their study, puerarin dose-dependently and circadian time-dependently induced the expression of NR1D1 target genes (*Bhmt*, *Cbs* and *Cth*, three enzymes involved in homocysteine decomposition) in HEPA-1C1C7 cells. These results suggest that puerarin alleviates methionine-induced hyperhomocysteinemia by targeting NR1D1. Conversely, this protective effect disappeared in NR1D1-deficient cells [87]. In a study on the dual roles of the circadian clock in regulating bilirubin detoxification, Wang et al. [111] demonstrated that bilirubin stimulated BMAL1 expression by antagonizing NR1D1, constituting a feedback mechanism for bilirubin detoxification. In summary, natural products have potential as regulators of circadian rhythms and provide a theoretical basis for the development of low-toxicity therapeutic drugs.

### Synthetic antagonists of NR1D1

SR8278 was the first identified synthetic antagonist of NR1D1 and was discovered by Kojetin and colleagues in 2010 [82]. GSK4112 and heme can both be rendered inactive by SR8278, but their exact binding location and level of competition with heme are still unknown [82]. As the most frequently used NR1D1 antagonist in preclinical models to date, SR8278 exerts anti-inflammatory, antifibrotic and metabolic regulatory effects [73, 112–114]. Multiple studies have confirmed that SR8278 has great potential as a therapeutic agent for neurodegenerative diseases such as epilepsy, Parkinson’s disease, and Alzheimer’s disease, and its specific pharmacological effects appear to be related to the chemical properties of the neuron (i.e., the type of neurotransmitter released at the neuronal endings) [115–117]. Paradoxically, SR8278 has unfavorable pharmacokinetics, such as a short half-life, large volume of distribution, and high clearance in rats, but shows excellent clinical potential in streptozotocin-induced diabetic rats [118]. The reason for this paradox may be related to the mode of action of SR8278 or different pathological backgrounds. However, there is no doubt that SR8278 may serve as a starting point for the development of NR1D1 antagonists with increased potency and effectiveness.

GSK1362 (GSK3201362) is an antagonist of NR1D1, and the interaction with the O-methyl ethanolamine side chain and Lys473 through hydrogen bonds is essential for its effects. In contrast to GSK4112, GSK1362 inhibits LPS-induced production of several inflammatory cytokines by alveolar macrophages, especially *Il-6* gene expression [119]. Notably, GSK1362 cannot currently be considered a chemical probe due to its poor predicted pharmacokinetic profile, unidentified other targets, and unidentified off-target effects [119].

These findings suggest that, although only effective in animal models, synthetic NR1D1 ligands that pharmacologically target the circadian rhythm may be helpful in many pathophysiological processes and the treatment of associated disorders. Additionally, despite the extensive establishment of the pharmacological effects of NR1D1 ligands on animals (preclinical investigations), no progress has been made in clinical trials. Poor pharmacokinetics caused by poor bioavailability are currently the key obstacles restricting the development and clinical use of NR1D1 antagonists. The short half-life allows the ligand to be rapidly cleared from mice after injection, and so multiple injections are required to ensure pharmacological activity. In addition, given the differences in circadian rhythms between humans and rodents, ligands that are effective in preclinical models may not necessarily be equally effective in clinical trials, and so exploring the pharmacological activity of novel ligands in primate models may be an important step in future preclinical trials.

## Summary and perspectives

As a vital transcriptional repressor, NR1D1 not only represses clock genes transcription, such as *Bmal1* and* Cry1*, to mediate circadian rhythms, but also plays critical roles in multiple pathophysiological processes of several organs, such as inflammation, autophagy, immunity, metabolism, and aging.

### The search for NR1D1 colocalized partners

The genetic program of any physiological or pathological process always involves the participation of multiple transcription factors (TFs) and chromatin remodeling factors. For example, the transcription factors P53, forkhead box class O protein (FOXO), Nrf2 and NF-kB all participate in the regulation of autophagy. Direct gene control has not resulted in clinically useful treatments, despite advances in our understanding of gene regulation and the proliferation of gene-targeted therapeutics. This is caused, at least in part, by the absence of instruments to coordinately alter several TFs in a spatiotemporal-specific way. Using heart tissue and ChIP-Seq, Zhang et al. [8] discovered that NR1D1 could colocalize with driving TFs and coordinate transcriptional repression at thousands of genomic loci controlled by several TFs, which can stop the pathologic transition of the gene program. However, only MEF2a and MEF2c were identified as colocalized partners for NR1D1 in the heart. Therefore, further knowledge about the full range of partners and targets of NR1D1 during pathological remodeling of the heart or other organs is needed, as well as the processes by which NR1D1 colocalizes with tissue-specific TFs and modifies the selective gene program throughout the disease process.

### The development of novel NR1D1 ligands

As described previously, NR1D1 physically binds to the promoter or enhancer regions of multiple genes and controls their expression, such as *Bmal1*, *Gata3*, and *Ulk1*, constituting a node-rich, functionally complex transcriptional regulatory network. To date, the most frequently used NR1D1 ligands are SR9009, GSK4112, and SR8278. Many NR1D1 ligands have been shown to target NR1D1 to induce pharmacological effects. Despite the pharmacological effects of NR1D1 ligands being well established in animals, there has been no advancement in their translation to human trials. The difficulties might include issues with stability and safety, poor pharmacokinetics, and potential differences in circadian systems between humans and rats/mice. As a result, it is advised that NR1D1 be targeted to treat local disorders and that a targeted treatment be administered locally to prevent negative effects on other tissues or organs. In addition, structural backbone-based drug design (e.g., exploring more efficient scaffold structures) and receptor dynamics-based drug screening (e.g., developing biased ligands) may also lead to new ideas for future therapeutic strategies targeting NR1D1.

### Exploration of rhythmic treatment strategies

Studies have reported circadian rhythms in some diseases, such as colitis, the severity of which is associated with circadian rhythms and opposite to NR1D1 expression [74], and the circadian biology of several main organ systems has been elucidated [120]. This suggests that future studies should attempt to identify key regulatory molecules (i.e., clock control checkpoints) controlled by the circadian clock during complex disease processes, thereby resulting in time-dependent strategies for diseases with clear rhythmic characteristics. It is also necessary to quickly elucidate the circadian biology of organ systems that have been poorly defined to date, including the reproductive system, circulatory system, and peripheral nervous system.

Melatonin affects the rhythmic regulation of NR1D1 expression, and in turn, alterations in clock gene rhythms play a role in melatonin production. First, in normal humans, the patterns of melatonin secretion and NR1D1 expression are broadly consistent with typical circadian patterns [121]. For example, nighttime lighting adversely affects melatonin secretion and NR1D1 expression. In addition, melatonin significantly inhibits receptor activator of nuclear factor-κB ligand (RANKL)-induced osteoclast formation in Raw264.7 cells by upregulating NR1D1 expression. Further studies confirmed that NR1D1 overexpression enhanced the anti-osteoclastogenesis effects of melatonin [122]. Moreover, in preeclamptic placental macrophages, melatonin production and oscillations were altered, and circadian rhythms regulated by clock genes were disrupted [123]. Currently, melatonin is widely used in the clinic [NCT03951025, NCT04229719, NCT02836743, NCT00692094], and in-depth studies of the link between melatonin and NR1D1 will provide potential therapeutic strategies for many diseases.

More importantly, even though the biological clock is based on a 24-h cycle, various species and even different organs and tissues/cells within the same organism have different biological clock cycles. For example, Formosan wood mice (WM) and laboratory C57BL/6 mice have variations in locomotor activity and sex variabilities. Shieh et al. [124] observed that male WM had more locomotor activity during the lights-out period than male C57BL/6 mice, but this effect was not observed in females. Moreover, sex differences in circadian rhythms have been observed in alpha murine urokinase-like plasminogen activator (αMUPA) transgenic mice and mice that were hemizygous for the 16p11.2 deletion (16p11.2 del/+). The former is evidenced by high-amplitude circadian rhythms with longer endogenous periods in female mice [125], and the latter has been shown by increased wakefulness and decreased non-rapid eye movement (NREM) sleep duration in male mice [126]. In addition, even cells originating from the same individual produce different rhythms over generations of inheritance. Kim et al. [127] demonstrated that skin fibroblasts from diversity outbred (DO) mice underwent increased variations in circadian phenotypes over the course of inheritance. However, the mechanism by which NR1D1 regulates circadian rhythms in different species has not yet been clearly explored. More research is still needed to determine the specific regulatory role of NR1D1 in other species.

## Conclusions

This review comprehensively summarizes the roles of NR1D1 in several vital organs, such as the heart, liver, lung, and kidney, by regulating key physiopathological processes, including autophagy, immunity, inflammation, metabolism, and aging. Given the pivotal biological functions of NR1D1, it is imperative to explore novel ligands targeting NR1D1 for the development of chemical probes and targeted drugs. Many NR1D1 ligands have been shown to improve inflammation, inhibit apoptosis, regulate metabolism and exert other biological effects in preclinical studies. However, there has been little clinical progress on NR1D1 ligands, and challenges include drug safety concerns, poor bioavailability and pharmacokinetic properties, and differences in circadian mechanisms between humans and rodents. Moreover, considering the intimate association between NR1D1 and the central circadian clock, NR1D1 also holds great promise for therapeutic strategies addressing rhythmic disorders.

## Data Availability

Data sharing is not applicable to this article as no new data were created or analyzed in this study.

## Abbreviations

AAA: Abdominal aortic aneurysm
AAI: Aristolochic acid I
AF-1: Activation function-1
AF-2: Activation function-2
AKI: Acute renal injury
ALD: Alcoholic liver disease
ALI: Acute lung injury
AMD: Age-related macular degeneration
Apo: Apolipoprotein
ATG5: Autophagy protein 5
Bmal1: Brain and muscle ARNT-like 1
BMDM: Bone marrow-derived macrophage
CCG: Clock-controlled gene
CLOCK: Circadian locomotor output cycles kaput
CRY: Cryptochrome
COPD: Chronic obstructive pulmonary disease
CS: Cigarette smoke
CYP: Cytochrome
CYP7A1: Cholesterol 7α-hydroxylase
DBD: DNA-binding domain
Dcg: D-box controlled gene
DENV: Dengue virus
dMφ: Decidual macrophage
EMT: Epithelial-mesenchymal transition
eRNA: Enhancer-derived RNA
ETC: Electron transport chain
E4BP4: E4 promoter binding protein 4
E75: Ecdysone-induced protein 75
FGF21: Fibroblast growth factor 21
FH: Fulminant hepatitis
FLS: Fibroblast-like synoviocyte
GATA3: GATA binding protein 3
GC: Granulosa cell
GEC: Gastric epithelial cell
GalN: Galactosamine
HCV: Hepatitis C virus
HDAC3: Histone deacetylase 3
HDL: High-density lipoprotein
hESC: Human endometrial stroma cell
HF: Heart failure
HFL-1: Human fetal lung fibroblast-1
HK1: Hexokinase 1
I/R: Ischemia/reperfusion
IMQ: Imiquimod
LBD: Ligand-binding domain
MD: Molecular dynamics
Mmp: Matrix metalloproteinase
MI: Myocardial infarction
MLF: Mouse lung fibroblast
mtDNA: Mitochondrial deoxyribonucleic acid
mPGES-2: Microsomal prostaglandin E synthase-2
NAFLD: Non-alcoholic fatty liver disease
NCOR: Nuclear receptor corepressor
NO: Nitric oxide
NR: Nuclear receptor
NR4A1: Nuclear receptor subfamily 4, group A, member 1
Nrf2: Nuclear factor erythroid 2-related factor 2
NR1D1: Nuclear receptor subfamily 1, group D, member 1
PARP: Poly-ADP-ribose polymerase
PCK1: Phosphoenolpyruvate carboxykinase 1
PDK4: Pyruvate dehydrogenase kinase 4
PER: Period
PPP: Pentose phosphate pathway
PKM2: Pyruvate kinase M2
PKA: Protein kinase A
RANKL: Receptor activator of nuclear factor-κB ligand
RCG: RORE/RevRE-controlled gene
RCC: Renal cell carcinoma
Stra8: Stimulated by retinoic acid gene 8
RORα: RAR related orphan receptor α
RORE/RevRE: ROR/REV-ERB-response element
SCD: Stearoyl-CoA-desaturase
SCN: Suprachiasmatic nucleus
SHP: Small heterodimer partner
Sod1: Superoxide dismutase 1
Serpina3: Serine protease inhibitory factor 3
SAEC: Small airway epithelial cell
SCLC: Small-cell lung cancer
TCA: Tricarboxylic acid
Th: T helper
THIQ: Tetrahydroisoquinoline
TF: Transcription factor
TFEB: Transcription factor EB
TFE3: Transcription factor E3
TTFL: Transcription-translation feedback loop
VSMC: Vascular smooth muscle cell
ULK1: Unc-51-like kinase 1
ZIKV: Zika virus
